# The curative effect analysis of simultaneous U-VATS for bilateral multiple primary early-stage lung cancers

**DOI:** 10.3389/fonc.2025.1639138

**Published:** 2025-08-04

**Authors:** Miao Shi, Long-fei Wang, Xue-chi Zhang, Li-wei Tang, Lei Zheng, Wen-tao Hu, Zhi-gang Liang

**Affiliations:** ^1^ Department of Thoracic Surgery, First Affiliated Hospital of Ningbo University, Ningbo, Zhejiang, China; ^2^ Ningbo University Schoool of Medicine, Ningbo, Zhejiang, China

**Keywords:** U-VATS, simultaneous resection, bilateral MPLCs, quality of life, perioperative outcomes

## Abstract

**Background:**

The incidence of multiple primary lung cancers (MPLCs) has been on the rise over the past decade, yet optimal surgical strategies remain debated. This study compared perioperative outcomes and long-term quality of life (QoL) between simultaneous and staged uniportal video-assisted thoracoscopic surgery (U-VATS) for bilateral early-stage MPLC.

**Methods:**

A retrospective cohort analyzed 69 patients undergoing simultaneous (n=28) or staged (n=41) U-VATS between March 2021 and December 2023. A comparative statistical analysis was conducted to assess perioperative efficacy and long-term QoL between simultaneous versus staged U-VATS in patients with bilateral synchronous MPLCs.

**Results:**

The simultaneous group exhibited smaller tumors (*P*=0.002) and included more smokers (*P*=0.019). Compared to staged surgery, simultaneous U-VATS resulted in a shorter hospital stay (8 vs. 14 days, *P*<0.001), reduced non-steroidal drug use (240 vs. 440 mg, *P*<0.001), and lower costs (CNY 41218.11 vs. CNY 68041.55, *P*<0.001), with comparable operative times (*P*=0.193). Pulmonary infections were less common following simultaneous surgery (3.6% vs. 24.4%, *P*=0.045). No 30-day mortality occurred. Longitudinal QoL assessment using a standardized 8-item symptom scale (cough, polypnea, pain, fatigue, sweating, insomnia, constipation, throat irritation) identified significant advantages for simultaneous surgery in polypnea (*P*=0.015) and pain control (*P*=0.013), whereas remaining symptoms showed comparable trajectories (all *P*>0.05).

**Conclusion:**

Simultaneous U-VATS may be a safe, cost-effective option for early-stage MPLC, particularly in patients with smaller tumors. Larger multicenter studies are warranted to validate these findings.

## 1 Introduction

In recent years, as U-VATS technology has become increasingly sophisticated, it has been widely used in clinical practice, becoming one of the common surgical methods (1). Its safety and efficacy have been confirmed in multiple clinical studies (2, 3). U-VATS offers core advantages such as less trauma, lighter pain, and faster recovery compared to the traditional multi-port method in bilateral lung cancer resection. Currently, most thoracic tumors can be surgically removed using U-VATS techniques, especially for lung cancer surgeries (1, 4, 5). Lung cancer is one of the most malignant tumors with the highest incidence and mortality rates globally, severely threatening human health. According to data released by the International Agency for Research on Cancer (IARC) under the World Health Organization (WHO) in GLOBOCAN 2022, there were approximately 2.481 million new cases of lung cancer worldwide in 2022, with a crude incidence rate of 31.5 per 100,000 (6). The Expert Consensus on Lung Cancer Screening in Asian Populations revealed significant differences between Asian and Caucasian patients in terms of epidemiology, smoking patterns, and driver gene mutation status. It recommends low-dose spiral CT (LDCT) for high-risk population screening (based on smoking history, intensity, family history, etc.), to be performed annually or biennially. Additionally, the consensus emphasizes the need to improve accessibility to lung cancer screening programs and enhance follow-up management capabilities. Establishing and implementing risk prediction models can further optimize the effectiveness of LDCT screening (7). In China, lung cancer has the highest incidence and mortality rates; in 2022, there were about 1.06 million new cases of lung cancer (8). Due to the rapid advancement of medical diagnostic technology, such as high-resolution thin-layer lung computed tomography(CT), an increasing number of patients are being diagnosed with multiple pulmonary nodules, leading to a gradual increase in the detection rate of bilateral MPLCs in clinical practice (9). MPLCs refer to the presence of two or more primary lesions in lung cancer patients, including synchronous MPLCs and metachronous MPLCs (10, 11). According to research reports from both domestic and international studies, synchronous MPLCs account for 0.8% to 14.5% of newly diagnosed lung cancers (12–14); metachronous MPLCs have a time-accumulation effect, with each lung cancer patient having a risk of 1% to 3% for developing a second primary lung cancer (15, 16). The incidence of MPLCs is closely related to factors such as smoking history and family history (17, 18).

Currently, there is no unified approach to the treatment of synchronous MPLCs, with surgical resection being the primary treatment method (19, 20). However, there is no consensus on which surgical approach to use and when to perform the surgery. With the increasing adoption of U-VATS and robotic surgery, the feasibility of simultaneous bilateral procedures has significantly improved. Studies indicate that concurrent surgeries can reduce total hospital stay duration and costs, mitigate the risk of tumor progression during the interval between separate surgeries, alleviate the psychological and physiological burdens of multiple surgeries on patients, and do not increase perioperative mortality rates. However, careful consideration is required for elderly patients or those with poor pulmonary function. In theory, simultaneous bilateral resection of synchronous MPLCs is the most ideal treatment model. However, due to the higher surgical risks and limited treatment experience associated with simultaneous bilateral resection, most medical centers opt for staged resection of synchronous MPLCs.

Current evidence regarding the safety and efficacy of U-VATS for simultaneous bilateral primary lung cancer remains limited. This study aims to further investigate the safety and efficacy of U-VATS for simultaneous bilateral primary lung cancer by comparing the outcomes and quality of life between concurrent and staged surgical approaches. The goal is to provide evidence-based support for the clinical feasibility of simultaneous U-VATS resection.

## 2 Materials and methods

### 2.1 Study subjects

**Figure 1:**
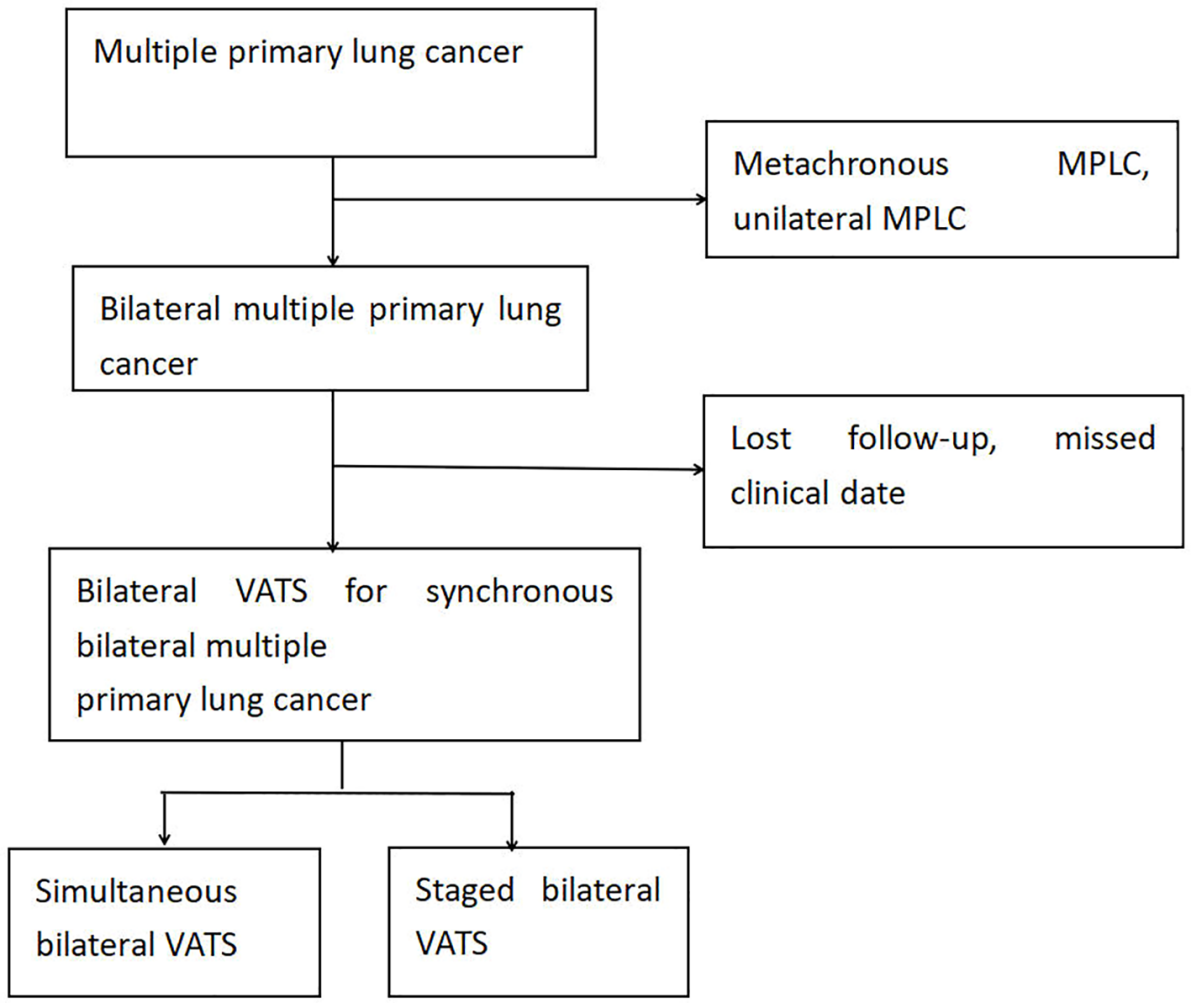
Flowchart of patient selection for this study. MPLC, multiple primary lung cancer; U-VATS, uniportal video-assisted thoracic surgery.

This study retrospectively collected clinical data of patients diagnosed with early-stage MPLCs (T1N0M0) post-thoracoscopic surgery at the Department of Thoracic Surgery, the First Affiliated Hospital of Ningbo University, from March 2021 to December 2023. A total of 156 patients with MPLCs were selected, including 69 patients who underwent bilateral thoracoscopic surgery, comprising 28 patients who received simultaneous bilateral thoracoscopic surgery and 41 patients who underwent staged bilateral thoracoscopic surgery. Simultaneous surgery refers to the resection of lesions in both lungs during the same anesthesia process within a single hospitalization period. For missing follow-up data, we employed the following analytical approaches: complete-case analysis was applied to primary outcome measures, while multiple imputation methods were utilized for secondary outcomes, supplemented by sensitivity analyses to compare differences between pre- and post-imputation results. Inclusion criteria were: ① Patients confirmed to have bilateral MPLCs post-surgery; ② Bilateral MPLCs were treated with thoracoscopic surgery; ③ All lung cancer patients had not received any adjuvant radiotherapy or chemotherapy prior to surgery;④Patient’s preoperative examination shows that arterial blood gas analysis with oxygen partial pressure and pulmonary function tests were normal. Exclusion criteria were: ①Malignant tumors involving other systems; ②Other lung diseases; ③Severe cardiac and pulmonary insufficiency; ④Failure to sign the informed consent for this study (**Figure 1**).

### 2.2 Preoperative assessment and surgical approach

All patients underwent evaluation of cardiac and pulmonary function upon admission, which included blood gas analysis, echocardiography, chest CT scans, pulmonary function tests, and brain magnetic resonance imaging (MRI). For solid nodules larger than 1 cm in diameter or mixed ground-glass nodules with a solid component larger than 1 cm, positron emission tomography CT (PET/CT) scans or bone scans were conducted to rule out distant metastasis.

The surgical method was tailored based on the tumor’s size, location, CT values, and consolidation tumor ratio(CTR), encompassing lobectomy, segmentectomy, and wedge resection, all in line with oncological principles. For patients with bilateral MPLCs undergoing simultaneous resection, the surgery commenced with the side that would incur less loss of lung function post-resection. The patient’s position was then altered to address the side with the more intricate or extensive resection (21). In the case of staged resection for bilateral MPLCs, the primary lesion (central type, rapid progression, large size, high solid component, and evident malignant signs) was removed first, followed by the secondary lesion (peripheral type, slow progression, small size) if the patient’s condition permitted (22). The surgical incision for this research is selected between the anterior axillary line and the middle axillary line at the 5th rib space, with the length of the surgical incision approximately 3 cm. The timing for the second surgery was determined by each patient’s recovery progress and postoperative thin-layer CT examination.

### 2.3 Postoperative follow-up

Follow-up data were obtained through telephone or first direct outpatient checks after surgery. According to the current cancer symptom assessment tools, clinical guidelines and expert interviews, we selected 8 core postoperative symptoms as an alternative item, including cough, polypnea, pain, fatigue, sweating, insomnia, constipation and throat irritation to assess the quality of life of patients during their follow-up examination three months after surgery. The degree of symptoms was assessed using the Numerical Rating Scale (NRS) on a scale of 0 to 10, with higher scores indicating more severe symptoms (23–25).

### 2.4 Statistical methods

Categorical variables are presented as counts and percentages. Continuous variables that follow a normal distribution are expressed as mean ± standard deviation, while those with skewed distributions are presented as median and interquartile range. Comparisons of continuous variables are performed using Student’s t-test or the Wilcoxon rank-sum test. For comparisons of age and total costs as well as postoperative symptom scores between two groups among continuous variables, the t-test is used; for other continuous variables, the rank-sum test is applied. Comparisons of categorical variables are conducted using Pearson’s chi-square test, Fisher’s exact test, or continuity correction. Additionally, multivariate logistic regression analysis was performed to assess baseline differences between the two groups. All statistical tests were two-sided with a significance level set at 0.05, utilizing the SPSS version 26.0 statistical software.

## 3 Results

### 3.1 Patient baseline characteristics

The basic information of the patients in both groups is shown in **Table 1**. There were 28 patients who underwent simultaneous bilateral thoracoscopic surgery, including 7 males and 21 females, with an average age of 50.39 ± 11.26. In contrast, there were 41 patients who underwent staged bilateral thoracoscopic surgery, including 5 males and 36 females, with an average age of 54.80 ± 8.56. There were no significant differences in gender, age, history of other diseases (including hypertension, diabetes, and cardiovascular diseases), number of lesions, or lung function at the time of the first surgery between the simultaneous and staged surgery groups. However, there was a significant difference in smoking history between the two groups (*P*=0.019), as well as a significant difference in the types of surgical combinations (*P*=0.008).

**Table 1:** Characteristics of the patients who underwent bilateral U-VATS for synchronous bilateral MPLCs.

Characteristics	Simultaneous bilateral U-VATS (n=28)	Staged bilateral U-VATS (n=41)	95%CI	*P* value
Age, years	50.39 ± 11.26	54.80 ± 8.56	[-0.35, 9.18]	0.069
Gender			[-1.17, 1.42]	0.292
Male	7 (25.0%)	5 (12.2%)		
Female	21 (75.0%)	36 (87.8%)		
Smoking history			[0.07, 0.18]	0.019
Yes	5 (17.9%)	0 (0)		
No	23 (72.1%)	41 (100%)		
Hypertension			[-0.32, 0.12]	0.507
Yes	6 (21.4%)	13 (31.7%)		
No	22 (78.6%)	28 (68.3%)		
Diabetes			[-0.03, 0.11]	0.847
Yes	1 (3.6%)	0 (0)		
No	27 (96.4%)	41 (100%)		
Cardiopathy			[-0.14, 0.08]	0.897
Yes	1 (3.6%)	3 (7.3%)		
No	27 (96.4%)	38 (92.7%)		
Number of lesions			[-0.06, 0.42]	0.138
2	16 (57.1%)	16 (39.0%)		
≥3	12 (42.9%)	25 (61.0%)		
Size of the largest lesions, mm	7 (6.00,8.75)	9 (7.50, 13.50)	[1, 4]	0.002
Pulmonary function at first operation				
FVC (mean,L)	2.89 (2.65, 3.22)	2.79 (2.44, 3.17)	[-0.36, 0.12]	0.300
FEV1 (mean,L)	2.43 (2.28, 2.64)	2.31 (1.99, 2.56)	[-0.35, 0.03]	0.096
FEV1%	97.35 (86.70, 101.48)	98.8 (89.95, 107.45)	[-4.00, 9.10]	0.457
Combination of surgical types			[0.94, 1.46]	0.008
Wedge-Wedge	7 (25.0%)	4 (9.8%)		
Wedge-Segment	17 (60.7%)	12 (29.3%)		
Wedge-Lobe	1 (3.6%)	7 (17.1%)		
Segment-Lobe	2 (7.1%)	8 (19.5%)		
Segment-Segment	1 (3.6%)	8 (19.5%)		
Lobe-Lobe	0 (0)	2 (4.9%)		

### 3.2 Surgical data

The perioperative surgical evaluation indicators for both groups of patients are shown in **Table 2**. The median surgery duration for the simultaneous thoracoscopic surgery group was 145 (115, 184) minutes, and for the staged thoracoscopic surgery group, it was 165 (120, 215) minutes, with no statistical difference between the two groups (*P*=0.193). The median hospital stay for patients in the simultaneous thoracoscopic surgery group was 8 (7, 9.75) days, and for the staged thoracoscopic surgery group, it was 14 (12, 17) days, showing a significant difference in hospital stay, with staged surgery patients having a longer hospital stay than simultaneous surgery patients (*P*<0.001). The median dosage of analgesic medication used by patients in the simultaneous thoracoscopic surgery group was 240 (240, 320) mg, and for the staged thoracoscopic surgery group, it was 440 (360, 560) mg, indicating a significant difference in the dosage of postoperative analgesic medication, with staged surgery patients using significantly more analgesic medication than simultaneous surgery patients (*P*<0.001). The median intraoperative blood loss for patients in the simultaneous thoracoscopic surgery group was 20 (10, 20) ml, and for the staged thoracoscopic surgery group, it was 40 (20, 60) ml, showing a significant difference in intraoperative blood loss, with staged surgery patients having more blood loss than simultaneous surgery patients (*P*<0.001). The median duration of postoperative chest tube placement for patients in the simultaneous thoracoscopic surgery group was 4 (3, 5) days, and for the staged thoracoscopic surgery group, it was 7 (6, 8) days, indicating a significant difference in the duration of postoperative chest tube placement, with staged surgery patients having a significantly longer duration than simultaneous surgery patients (*P*<0.001). The average hospitalization cost for patients in the simultaneous thoracoscopic surgery group was 41218.11 ± 10308.12 CNY, and for the staged thoracoscopic surgery group, it was 68041.55 ± 12797.21 CNY, showing a significant difference in hospitalization costs, with staged surgery patients having significantly higher hospitalization costs than simultaneous surgery patients (*P*<0.001). However, there was no significant difference in the 30-day postoperative mortality rate between the two groups, both being 0. This is likely attributable to the fact that all enrolled patients in this study were in early-stage disease (T1N0M0) and those with cardiopulmonary insufficiency were excluded.

**Table 2:** Perioperative outcomes of bilateral MPLCs.

Parameter	Simultaneous bilateral U-VATS (n=28)	Staged bilateral U-VATS (n=41)	95%CI	*P* value
Total operative time, min	145 (115,184)	165 (120, 215)	[-10, 50]	0.193
Total hospital stays, day	8 (7, 9.75)	14 (12, 17)	[5, 7]	<0.001
Total dose of painkillers, mg	240(240, 320)	440 (360, 560)	[120, 200]	<0.001
Total blood loss, ml	20 (10,20)	40 (20, 60)	[10,35]	<0.001
Total drainage time, day	4 (3, 5)	7 (6, 8)	[2, 4]	<0.001
Total cost, CNY	41218.11 ± 10308.12	68041.55 ± 12797.21	[21021.19, 32625.70]	<0.001
30-day mortality, n (%)	0 (0.0)	0 (0.0)	–	–

CNY, Chinese Yuan.

### 3.3 Postoperative complications

The postoperative complication rates for both groups of surgical patients are shown in **Table 3**. In the simultaneous thoracoscopic surgery group, there was 1 case of postoperative pulmonary infection (3.6%), while in the staged thoracoscopic surgery group, there were 10 cases of postoperative pulmonary infection (24.4%), indicating a significant difference between the two groups (*P*=0.045). There were no significant differences in postoperative complications such as air leak, postoperative cerebral infarction, and incision infection between the simultaneous thoracoscopic surgery group and the staged thoracoscopic surgery group. Additionally, neither group experienced respiratory failure, pulmonary embolism, or postoperative death.

**Table 3:** Postoperative complications after simultaneous and staged bilateral U-VATS.

Complications	Simultaneous bilateral U-VATS (n=28)	Staged bilateral U-VATS (n=41)	*P* value
Pulmonary infection, n(%)	1 (3.6%)	10 (24.4%)	0.045
Air leakage, n(%)	3 (10.7%)	7 (17.1%)	0.465
Respiratory weakness, n(%)	0 (0.0)	0 (0.0)	–
Postoperative bleeding, n(%)	0 (0.0)	0 (0.0)	–
Pulmonary embolus, n(%)	0 (0.0)	0 (0.0)	–
Cerebral infarction, n(%)	0 (0%)	1 (2.4%)	1.000
Wound infection, n(%)	1 (3.6%)	2 (4.9%)	0.795

### 3.4 Long-term quality of life assessment after surgery for lung cancer patients

**Figure 2:**
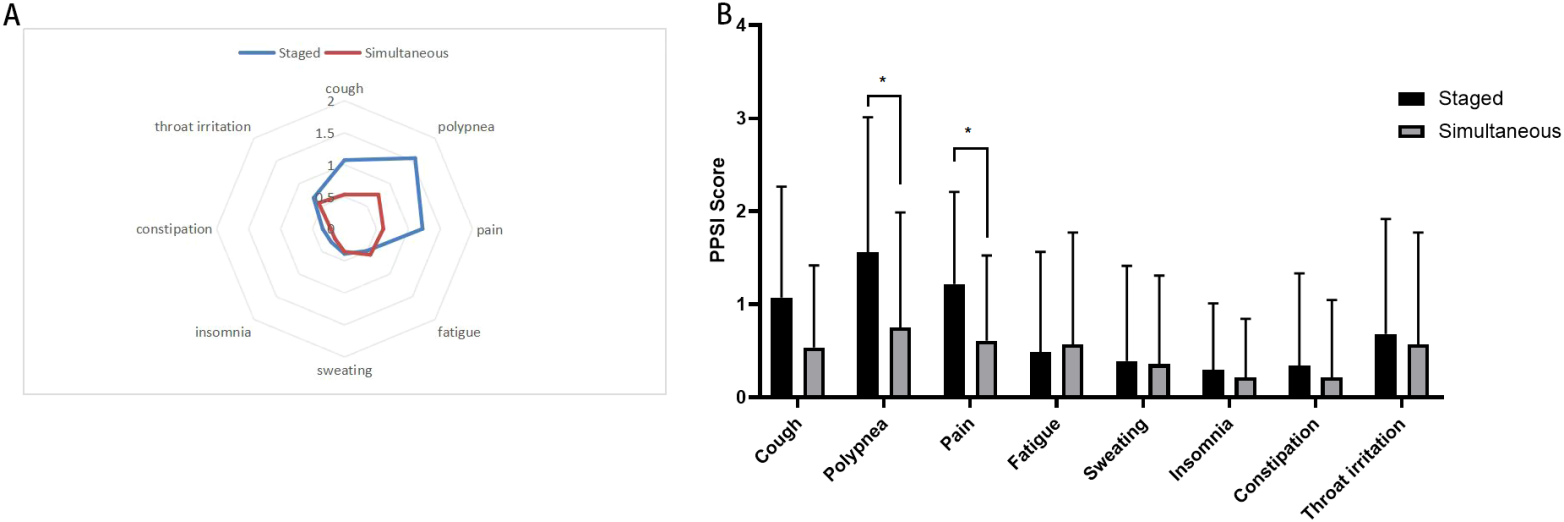
Postoperative symptoms score after simultaneous and staged bilateral U-VATS, **(A)** Radar map displays the mean postoperative symptom scores between two groups. Each axis of the radar chart represents a distinct symptom, with scores increasing radially from the central point “asymptomatic” toward the outermost circumference “most severe symptoms”; **(B)** Bar chart: presents postoperative symptom scores between two groups. The X-axis represents eight postoperative symptoms, while the Y-axis indicates symptom severity scores (e.g., 0–10 Visual Analog Scale). *indicates a statistically significant difference in symptom scores between the two groups (P < 0.05).

The long-term quality of life assessment for both groups of surgical patients is shown in **Figure 2** and **Table 4**. According to the current cancer symptom assessment tools, clinical guidelines and expert interviews, we selected 8 core postoperative symptoms, such as cough, polypnea, pain, fatigue, sweating, insomnia, constipation and throat irritation, to assess the quality of life of patients during their follow-up examination three months after surgery. There were no cases of severe postoperative symptoms at the incision site in either group, but there were significant difference in polypnea and pain between the two groups (*P*=0.015 and *P*=0.013). This may be due to the fact that simultaneous surgery requires only one anesthesia and postoperative recovery process, which reduces the impact of repeated trauma on the body. Staged surgery necessitates a second operation to resect lung tissue again, leading to further impairment of pulmonary function. Compared to staged surgery, simultaneous surgery results in milder postoperative pain and polypnea. However, there were no significant difference in the another postoperative symptoms,including cough, fatigue, sweating, insomnia, constipation and throat irritation.

**Table 4:** Postoperative common clinical symptom scores at 3 months for simultaneous and staged bilateral U-VATS.

Symptoms	Simultaneous bilateral U-VATS	Staged bilateral U-VATS	95%CI	P value
Cough	0.98 ± 0.86	1.07 ± 1.19	[-0.01, 1.06]	0.059
Polypmea	0.75 ± 1.24	1.56 ± 1.45	[0.14, 1.48]	0.018
Pain	0.61 ± 0.92	1.21 ± 0.99	[0.14, 1.08]	0.011
Fatigue	0.57 ± 1.20	0.49 ± 1.08	[-0.64, 0.47]	0.763
Sweating	0.36 ± 0.95	0.39 ± 1.02	[-0.45, 0.52]	0.892
Insomnia	0.21 ± 0.63	0.29 ± 0.72	[-0.26, 0.41]	0.641
Constipation	0.21 ± 0.83	0.34 ± 0.99	[-0.31, 0.57]	0.566
Throat irritation	0.57 ± 1.20	0.68 ± 1.23	[-0.48, 0.71]	0.711

### 3.5 Multivariate logistic regression analysis of postoperative pulmonary infection in patients undergoing U-VATS in both simultaneous and staged procedures

Age, smoking status, maximum tumor size, and surgical types were included in the multivariate logistic regression model. The results showed that age, smoking status, maximum tumor size, and surgical types did not increase the risk of postoperative pulmonary infection, and there was no statistical significance (**Table 5**).

**Table 5:** A multivariate binary logistic regression analysis for pulmonary infection after simultaneous and staged bilateral U-VATS.

Variable	B value	Odds Ratio(OR)	95%CI for OR	*P*-value
Smoking	-19.31	0	[0, -]	0.999
Size of the largest lesions	0.15	1.16	[0.97, 1.38]	0.112
Age	0.01	1.01	[0.93, 1.10]	0.664
Surgical Types	0.13	1.13	[0.67, 1.93]	0.643

## 4 Discussion

The evolution of thoracoscopic techniques has seen significant advancements, moving from traditional multiportal methods to single-utility-port setups, and now predominantly featuring (26, 27) U-VATS. According to relevant literature reports, the long-term efficacy of U-VATS shows no significant difference compared with traditional open thoracotomy and multiportal VATS (5, 28, 29). This trajectory underscores the surgical community’s commitment to minimizing invasiveness while maximizing patient-centered outcomes. Concomitantly, advancements in low-dose spiral CT coupled with AI-assisted screening have substantially enhanced early detection rates of MPLCs (30). Nevertheless, persistent heterogeneity in diagnostic interpretation and therapeutic strategies among clinicians continues to impede standardized management protocols (31). Our study’s theoretical framework was established through rigorous integration of the Martini-Melamed criteria and the American College of Chest Physicians guidelines, addressing this critical knowledge gap (32).

The optimal sequencing of bilateral U-VATS for MPLCs remains clinically contested. Our retrospective analysis of 69 patients undergoing U-VATS revealed critical insights. While simultaneous resection theoretically offers dual therapeutic advantages—single-anesthesia completion and avoidance of secondary surgical stress—it necessitates meticulous patient selection predicated on lesion topography, cardiopulmonary reserve, and surgical expertise. Current clinical paradigms preferentially target younger, non-emphysematous patients, though formal consensus guidelines remain elusive. Our institutional protocol mandated stringent preoperative cardiopulmonary functional assessments (normoxic arterial blood gases, preserved spirometry) and functional capacity validation (≥8-flight stair climb).

Notably, our cohort demonstrated superior perioperative safety profiles compared to historical data: 30-day mortality was null, with postoperative complications occurring in only 17.86% (5/28) of simultaneous resection cases—below the 21.95% benchmark reported by Hui Zheng et al (22). Longitudinal follow-up incorporating structured patient-reported outcomes (PROs) revealed comparable chronic incision-related morbidity between simultaneous and staged approaches, with suggestive trends favoring simultaneous resection in six-month dyspnea indices. These findings align with Mun et al.’s (33) documentation of satisfactory outcomes in 14 bilateral U-VATS cases, reinforcing the viability of this strategy in appropriately selected populations.

In patients with bilateral MPLCs, the postoperative rate of pulmonary infection is higher in those undergoing staged surgery compared to those undergoing simultaneous surgery. This may be attributed to the compounded effects of surgical trauma and exacerbated immunosuppression (34). Staged surgery necessitates two separate anesthetic events and surgical interventions, each of which can activate systemic inflammatory responses (e.g., increased levels of IL-6 and TNF-α) and suppress immune function (e.g., reduction in CD4+ T cells and decreased NK cell activity) (35, 36). Although simultaneous surgery involves a greater degree of trauma in a single procedure, it only subjects the patient to one period of immunosuppression, allowing for faster postoperative immune recovery and relatively lower infection risk. During the interval between two staged surgeries, patients may remain in a state of persistent immunosuppression, increasing the risk of opportunistic infections (such as Gram-negative bacteria and Staphylococcus aureus) (37, 38). Additionally, patients undergoing staged surgery may experience two separate incision sites leading to pain, which can result in restricted breathing and ineffective coughing, thus increasing the risk of atelectasis and further pulmonary infection.

This study found that in patients with bilateral primary lung cancer, staged surgery resulted in more significant postoperative pain and polypnea compared to simultaneous surgery. This may be due to the fact that simultaneous surgery requires only one anesthesia and postoperative recovery process, reducing the impact of repeated trauma on the body, thus resulting in milder postoperative pain compared to staged surgery (36, 39). In patients undergoing staged surgery, after the first operation, part of the lung tissue has been resected, and the remaining lung tissue needs compensatory expansion to maintain respiratory function. If lung tissue is resected again during the second surgery, lung function is further impaired, leading to more pronounced postoperative polypnea (40). On the other hand, although simultaneous surgery involves a larger resection range at once, the remaining lung tissue can gradually adapt through early rehabilitation training, avoiding the problem of insufficient functional compensation between the two surgeries, hence, simultaneous surgery results in milder postoperative polypnea compared to staged surgery.

While Simultaneous surgery can shorten total hospitalization duration and reduce costs, avoid tumor progression risk during the interval period of staged procedures, decrease postoperative infection rates, pain and dyspnea complications, without increasing perioperative mortality, it still requires cautious evaluation for elderly patients or those with compromised pulmonary function. Although this technique is minimally invasive with rapid recovery, overdiagnosis and overtreatment risks should be cautiously considered for pure GGNs ≤1 cm. Current evidence shows that 18%-35% of pure GGNs <5 mm remain stable long-term, thus we recommend confirming their growth patterns through at least 2 years of dynamic CT surveillance (with particular attention to indolent nodules exhibiting volume doubling times >400 days) (41–44).

This investigation acknowledges critical constraints: 1) limited cohort size and intermediate follow-up duration necessitate large-scale validation; 2) potential selection bias inherent to retrospective designs; 3) absence of oncological recurrence and survival data. All patients were treated at a single institution, potentially introducing selection bias due to localized referral patterns and surgeon-specific preferences. Additionally, the modest sample size (n=69) may underpower subgroup analyses and limit generalizability. Multicenter collaborations with larger cohorts are necessary to confirm these preliminary findings. This study lacks long-term survival and recurrence data, and therefore cannot elucidate differences in long-term survival rates between the two groups of patients. The enrolled patients in this study exhibited certain baseline imbalances, which may impact the clinical applicability of our findings. To address these confounding factors, we initially employed propensity score matching (PSM) for adjustment (45). However, through in-depth analysis, we identified that the current sample size (N=69) limited the effective application of PSM. To mitigate selection bias, we further incorporated multivariate logistic regression analysis, adjusting for key confounding variables including tumor size and smoking history (**Table 5**). To enhance the reliability and clinical applicability of the current study’s conclusions, the next step will involve conducting multicenter prospective clinical research. The focus will be on addressing existing limitations such as single-center bias, missing survival data, and insufficient validation of standardized patient-reported outcome (PRO) tools through matched cohort design or randomized controlled trials.

In summary, U-VATS emerges as a strategically sound modality for simultaneous bilateral resection of early-stage MPLCs in optimized candidates. This paradigm synergistically achieves dual objectives: preserving postoperative quality of life while demonstrating significant hospital cost containment (mean CNY 41218.11 vs. CNY 68041.55 for staged approaches, P<0.001)—a critical consideration in value-based healthcare models. Our findings advocate for broader adoption of this approach to optimize healthcare resource utilization without compromising therapeutic integrity.

## Data Availability

The original contributions presented in the study are included in the article/supplementary material. Further inquiries can be directed to the corresponding authors.
